# Carbamazepine Reduces Sharp Wave-Ripple Complexes and Exerts Synapse-Specific Inhibition of Neurotransmission in Ex Vivo Hippocampal Slices

**DOI:** 10.3390/brainsci11060787

**Published:** 2021-06-15

**Authors:** Timothy A. Simeone, Segewkal H. Heruye, Joseph A. Kostansek, Mary Y. Yeh, Stephanie A. Matthews, Kaeli K. Samson, Kristina A. Simeone

**Affiliations:** Department of Pharmacology & Neuroscience, Creighton University School of Medicine, Omaha, NE 68174, USA; SegewkalHeruye@creighton.edu (S.H.H.); JoeKostansek@creighton.edu (J.A.K.IV); MaryElizabethYeh@creighton.edu (M.Y.Y.); StephanieMatthews@creighton.edu (S.A.M.); KaeliSamson@creighton.edu (K.K.S.); kristinasimeone@creighton.edu (K.A.S.)

**Keywords:** cognitive impairment, epilepsy, adverse effect, high frequency oscillations

## Abstract

Higher therapeutic concentrations of the antiseizure medication carbamazepine (CBZ) are associated with cognitive side effects. Hippocampal sharp wave-ripple complexes (SPW-Rs) are proposed to participate in memory consolidation during periods of quiet and slow-wave sleep. SPW-Rs are generated in the CA3 region and are regulated by multiple synaptic inputs. Here, we used a multi-electrode array to determine the effects of CBZ on SPW-Rs and synaptic transmission at multiple hippocampal synapses. Our results demonstrate that CBZ reduced SPW-Rs at therapeutically relevant concentrations (IC_50_ = 37 μM) and altered the core characteristics of ripples, important for information processing and consolidation. Moreover, CBZ inhibited neurotransmission in a synapse-specific manner. CBZ inhibition was most potent at the medial-perforant-path-to-CA3 and mossy-fiber-to-CA3 synapses (IC_50_s ~ 30 and 60 μM, respectively) and least potent at medial-perforant-path-to-dentate granule cell synapses (IC_50_ ~ 120 μM). These results suggest that the synapse-specific CBZ inhibition of neurotransmission reduces SPW-Rs and that the CBZ inhibition of SPW-Rs may underlie the cognitive impairments observed with therapeutic doses of CBZ.

## 1. Introduction

Carbamazepine (CBZ) is an oral anti-seizure medication that is also effective in the treatment of bipolar disorder and trigeminal neuralgia [1]. CBZ has been frequently reported to adversely affect cognitive function, although contrary findings exist [2,3,4]. These conflicting results most likely stem from the dose-dependent effects of CBZ in humans, especially on attention, memory, learning, and language processing [4,5].

The primary mechanism of action for CBZ involves stabilizing voltage-dependent sodium channels in a fast-inactivated state, resulting in the inhibition of neuronal activity in a use-dependent fashion [6]. Depending on the CBZ concentration, this activity-dependent inhibition allows normal, non-pathological action potentials to occur while blocking high-frequency firing associated with epileptiform activity or pain signals [6]. Presumably, therapeutic concentrations have little effect on normal neuronal signaling, whereas higher concentrations would result in cognitive side effects. However, certain forms of memory processing involve the high-frequency activity of neurons/networks. Indeed, CBZ blocks in vivo and ex vivo hippocampal long-term potentiation (LTP) [7]. Network oscillations generated in the hippocampus also play an important role in memory consolidation [8]. These oscillations include sharp waves (SPWs) that are often accompanied by high-frequency oscillation (HFO) complexes [8]. Memory-associated HFOs occur in the ripple band (80–200 Hz), are generated in the CA3 region of the hippocampus, and spread to para-hippocampal regions during rest and slow-wave sleep. Spontaneous SPW-ripples occur in ventral hippocampal slice preparations ex vivo, which permit the investigation of mechanisms underlying these network activities and the effects of therapeutic drugs [9,10,11]. A previous study found that the effects of the anesthetic/amnesic thiopental and the antiseizure medication phenobarbital on ex vivo SPW-ripples correlated with clinical effects on memory [9]. Here, we demonstrate for the first time that CBZ reduces hippocampal SPW-ripples at clinically relevant concentrations. Furthermore, the potency of CBZ’s effects on neurotransmission was synapse specific.

## 2. Materials and Methods

C3HeB/FeJ mice were purchased from Jackson Laboratories (Bar Harbor, Maine) and a colony was maintained in the Animal Resource Facilities at Creighton University School of Medicine. Mice were given food and water ad libitum and kept on a 12-h light/dark cycle. All procedures were in accordance with National Institutes of Health guidelines, EU Directive 2010/63/EU, and were approved by the Institutional Animal Care and Use Committee at Creighton University School of Medicine.

Mice aged 31–45 postnatal days were anesthetized with isoflurane, decapitated, and their brains were removed and quickly placed into ice-cold oxygenated (95% O_2_/5% CO_2_) cutting artificial cerebrospinal fluid (aCSF) containing (in mM): 206 sucrose, 2.8 KCl, 1 CaCl_2_, 1 MgCl_2_, 2 MgSO_4_, 26 NaHCO_3_, 1.25 NaH_2_PO_4_, and 10 glucose (pH 7.4). Horizontal sections (350 μM) of ventral hippocampal-entorhinal cortex (HEC) were cut on a Leica VT1200 (Leica Microsystems Inc., Bannockburn, IL, USA) and transferred to a holding chamber for 1 h containing warm (32 °C), oxygenated recording aCSF containing (in mM): 125 NaCl, 3.0 KCl, 2.4 CaCl_2_, 2.5 MgSO_4_, 26 NaHCO_3_, 1.25 NaH_2_PO_4_, and 10 glucose (pH 7.4). An HEC slice was placed on the array such that the microelectrodes spanned from CA1 to CA2, and the dentate gyrus to CA3 [10]. Twenty slices from fifteen mice were used for this study. A custom probe cap allowed for the delivery of humidified air (95% O_2_/5% CO_2_) and the perfusion (1 mL min^−1^) of in-line pre-warmed oxygenated aCSF that resulted in a solution inlet temperature of ~33.2 °C and an outlet temperature of ~31.6 °C. The bath level was maintained near the interface.

Paired stimulations with varying inter-stimulus intervals were used to examine neurotransmission at Schaffer collateral (SC)–CA3, SC–CA1, mossy fiber (MF)–CA3, medial perforant path (MPP)–dentate gyrus (DG), lateral perforant path (LPP)–DG, MPP–CA3, and LPP–CA3 synapses. An electrode located in CA1 stratum radiatum (sr) was chosen for stimulations of the SC, and electrodes in CA1sr and CA3sr were chosen to analyze orthodromic SC–CA1 responses and antidromic SC–CA3 responses. MFs were stimulated with an electrode located in either the DG granule cell body layer or the DG hilar region, and responses were recorded in the CA3 stratum lucidum and CA3sr. The MPP was stimulated with an electrode located in the inner/middle molecular layer of the DG and identified by the elicitation of a prominent paired pulse depression in the dentate molecular layers. An electrode in the outer molecular layer/stratum lacunosum moleculare was used to stimulate the LPP and identified by paired pulse facilitation of responses in the DG molecular layers. Usually, multiple synaptic pathways were examined in each slice. Once field potential slopes (10–90%) stabilized, an input–output curve was generated, stimulation intensities were set to elicit 50% of the maximum response, and paired stimulations of increasing intervals were performed. Spontaneous activity was then obtained in a five minute gap-free recording. Subsequently, the perfusion medium was changed to CBZ-aCSF (1–300 μM diluted from a 100 mM stock in DMSO). When the peak effect of CBZ on evoked responses was obtained, spontaneous activity was recorded again. Two to three CBZ concentrations were successively applied to each slice. Recordings were acquired at a 20 kHz sampling rate with a bandwidth of 0.1 Hz–10 kHz using Mobius software (Witwerx Inc., Tustin, CA, USA).

Data were imported into Spike2 (v6) software (Cambridge Electronic Design, Cambridge, England). To identify DC shifts of the SPWs, raw recordings were subjected to a 50 Hz low-pass filter (−3 dB point = 70 Hz) using a finite impulse response (FIR) filter (1319 filter coefficients) and down-sampled to 2.5 kHz. Automated threshold detection of SPWs was set at 3 × peak-to-peak noise level. To visualize ripples, recordings were filtered with an FIR 100–175 Hz band-pass filter (−3 dB points = 80 Hz and 200 Hz; 1319 filter coefficients) and time-frequency representations generated with short-time Fourier transform (mean detrending, Hanning windowing, and an FFT size of 2048 points). To identify and quantify ripples, the root mean square (RMS) of the noise was calculated using a 3 ms sliding window. Automated threshold detection was set at 3–4 times the RMS standard deviation. The Spike2 script Burst_1.29.s2s was used to identify ripple bursts and required at least three consecutive cycle troughs with inter-trough intervals no greater than 30 ms in duration (≥33 Hz).

All data are reported as the mean ± standard error. Statistical significance was determined with paired *t*-tests of drug concentration vs. baseline, unless otherwise specified, using Prism software (v7, GraphPad Software, San Diego, CA, U.S.A.). IC_50_s were calculated using the Prism 7 log(inhibitor) vs. normalized response equation of form Y = 100/(1 + 10^(X-LogIC50)^) with a Hill slope constrained to −1. All chemicals and drugs were purchased from Sigma-Aldrich (St. Louis, MO, USA).

## 3. Results and Discussion

SPW-ripples are important for memory consolidation, as each individual ripple cycle encodes information. During learning tasks, SPW-ripple amplitude, frequency, and cycle duration increase [12,13]. Elimination of SPW-ripples results in impaired spatial memory recall, and a combination of decreased SPW-ripple incidence and increased intra-ripple frequency is associated with age-related cognitive decline [14,15,16].

**Figure 1:**
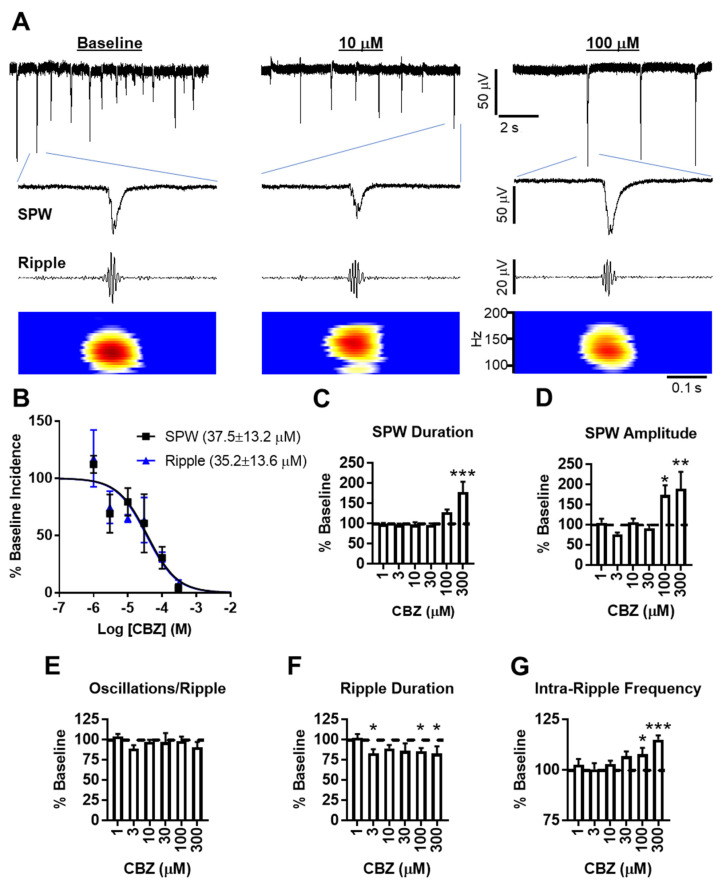
CBZ inhibits SPW-ripples. (**A**) Examples of SPW-ripple complexes recorded in the CA3 stratum radiatum at baseline and in the presence of CBZ. SPWs are evident in the broad band recordings (top traces), whereas ripples are fully identified via band-pass filtering and time frequency analyses (middle, bottom traces). (**B**) CBZ reduces the incidence of SPWs and ripples in a concentration-dependent manner. (**C**–**G**) High concentrations of CBZ significantly increase SPW duration, amplitude, and intra-ripple frequency, but CBZ has no effect on intra-ripple oscillations or duration (*n* = 5–8 slices per CBZ concentration; * *p* < 0.05, ** *p* < 0.01, *** *p* < 0.001 vs. baseline).

The baseline incidence of SPWs was 2.5 ± 0.3 s^−1^ (*n* = 17 slices) and was sensitive to CBZ inhibition, with an IC_50_ of ~37 μM and near-complete inhibition by 300 μM (Figure 1A,B). Average baseline SPW amplitudes and durations were 44 ± 12 μV and 56 ± 5 ms, respectively. SPWs remaining at 100 and 300 μM CBZ had significantly larger amplitudes and longer durations, suggesting that network events involving small populations are more sensitive to CBZ inhibition compared to triggers initiating the synchronous activity of large-population events (Figure 1A,C,D).

Band-pass filtering of the recordings and time frequency analyses with a short-time fast Fourier transform revealed the co-occurrence of ripples (Figure 1A), with ~44% of SPWs resulting in a ripple incidence of 1.1 ± 0.25 s^−1^. CBZ inhibited ripple incidence in a concentration-dependent manner, similar to SPWs with an IC_50_ of ~35 μM (Figure 1B). The examined ripple characteristics included oscillations/ripple, duration, and intra-ripple frequency, with average baseline measurements of 4.3 ± 0.2, 34 ± 2 ms, and 139 ± 4 Hz, respectively. CBZ had no effect on the number of oscillations during ripples, but there was a significant reduction in ripple duration and a significant increase in intra-ripple frequency by 100 and 300 μM CBZ (Figure 1E–G). Even though there appears to be an association between the intra-ripple frequency and SPW amplitude (Figure 1D,G), these two parameters were not correlated (nonparametric Spearman correlation, r = 0.111, *p* = 0.46), similar to previous findings for CA3 SPW-Rs recorded in vivo [17]. Rather, CBZ’s reduction of ripple duration compacted oscillations into a shorter timeframe, which resulted in the increase of intra-ripple frequency. The importance of this is supported by recent evidence demonstrating that short-duration ripples convey chunks of information (i.e., bits of a whole), whereas longer-duration ripples convey more complete information and improve working memory performance by providing time to recruit larger and more diverse populations of participating neurons [13]. Therefore, our current interpretation is that high concentrations of CBZ inhibit all but the strongest impulses to generate SPWs, resulting in a bias for larger amplitudes. At the same time, CBZ reduces SPW-induced neuronal firing and participation in ripples, resulting in shortened durations.

The calculated IC_50_s for SPW-ripples are well within the CBZ clinical plasma concentration target range of 4–12 mg/L, or 17 to 51 µM [18]. The brain-to-plasma ratio of CBZ is 1.5, indicating a therapeutic concentration of 25.5 to 76.5 μM in the temporal lobe. According to our results, therapeutic concentrations of CBZ would reduce the incidence of SPW-ripples by ~40–65%, whereas the characteristics that presumably convey information for consolidation are only affected by supratherapeutic concentrations of CBZ. Thus, therapeutic concentrations of CBZ may slow cognitive function, and supratherapeutic concentrations may be detrimental to the formation of memories. Similar alterations to ex vivo hippocampal SPW-ripples have been reported for sedative/amnesic concentrations of thiopental and phenobarbital, supporting the interpretation that CBZ’s effects on SPW-ripples have mechanistic relevancy to clinical adverse cognitive effects [9].

**Figure 2:**
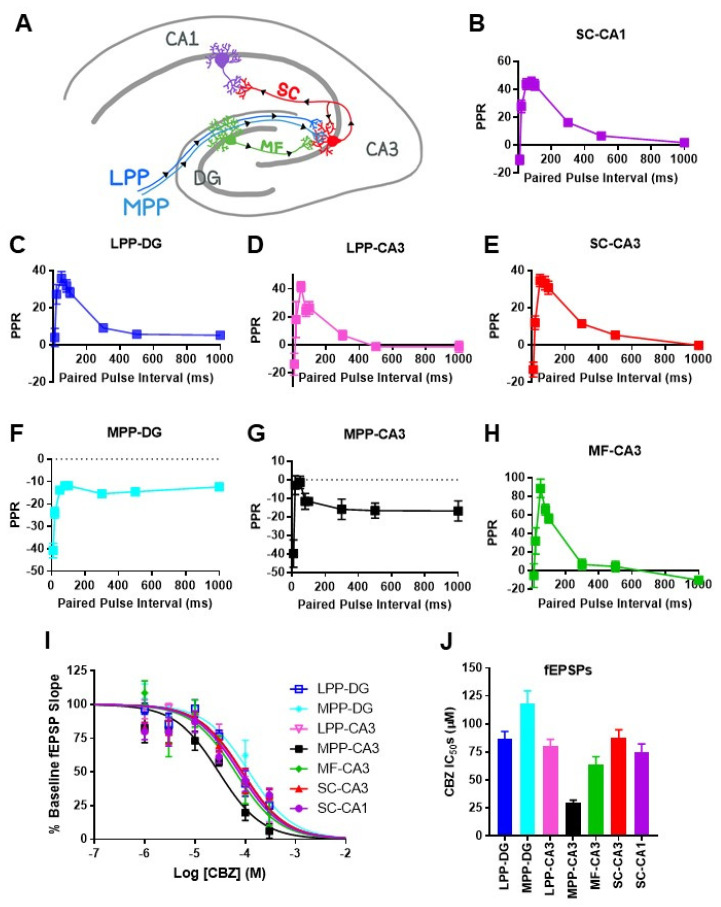
CBZ inhibits neurotransmission at multiple hippocampal synapses. (**A**) Illustration of hippocampal excitatory pathways and inputs from the entorhinal cortex via the lateral and medial perforant paths. (**B**–**H**) Identification of different synapses by characteristic short-term plasticity of excitatory postsynaptic field potentials (fEPSPs) to paired stimulations separated by increasing stimulations. (**I**) CBZ reduces fEPSPs at the seven synapses in a concentration-dependent manner. (**J**) Calculated half-maximal inhibitory concentrations (IC_50_s) from the concentration–response curves in I. IC_50_s: LPP–DG, 86.7 ± 7.6; MPP–DG, 118.3 ± 12.6; LPP–CA3, 80 ± 7.2; MPP–CA3, 29.5 ± 2.9; MF–CA3, 63.5 ± 8.3; SC–CA3, 87.5 ± 8.3; SC–CA1, 74.8 ± 8.4 (*n* = 4–7 slices per CBZ concentration).

Networks of pyramidal cells and interneurons in the CA3 region are theorized to generate SPW-ripples with inputs from dentate granule cell mossy fibers and entorhinal perforant path axons, modulating the SPW-ripple incidence and architecture [8,10]. SPW-ripple propagation is critically dependent on CA3 outputs [19]. To determine whether CBZ exerted synapse-specific effects, four fiber paths were stimulated, and seven regional synaptic responses were examined (Figure 2A). Synapses were identified by the placement of the stimulating and recording electrodes and characteristic plasticity responses to varying paired pulse intervals (Figure 2B–H). Synapses experiencing facilitation had maximal facilitation with a 50 ms interval between stimulations and a rank order of MF–CA3 > SC–CA1 > SC–CA3 = LPP–CA3 = LPP–DG, whereas paired pulse depression occurred at MPP–DG and MPP–CA3 synapses. The effect of CBZ on neurotransmission reduced the slopes of the evoked field potentials at all synapses. The IC_50_s for most synapses were in the high to supratherapeutic range, whereas the IC_50_ for the MPP–CA3 synapse was similar to SPW-ripples at ~30 μM, followed by the MF–CA3 synapse at ~64 μM (Figure 2I,J). Comparatively, there was a small range of CBZ sensitivities between synapses, with the most sensitive to least sensitive IC_50_ rank order of MPP–CA3 > MF–CA3 > SC–CA1 > SC–CA3 = LPP–DG = LPP–CA3 > MPP-DG. Accordingly, in the therapeutic range (25.5–76.5 μM), CBZ inhibited MPP–CA3 by 46–72%, MF–CA3 by 29–55%, SC–CA1 by 26–50%, SC–CA3 by 23–47%, LPP–DG by 23–47%, LPP–CA3 by 24–49%, and MPP–DG 18–39%, suggesting strong effects on neurotransmission at MPP-CA3 synapses and lesser effects on the other synapses. At the 300 μM supratherapeutic concentration, differences in inhibitory efficacy were more apparent, with 94%, 80%, 67%, 67%, 75%, 78%, and 77% inhibition, respectively.

In conclusion, our results indicate that SPW-ripple complexes are generated in the hippocampal CA3 region and regulated by mossy fiber and entorhinal inputs. We found that CBZ reduced SPW-ripple complexes in association with the preferential reduction of medial perforant-path- and mossy-fiber-to-CA3 neurotransmission. These synapse-specific effects of CBZ warrant future mechanistic investigations. CBZ inhibition of SPW-ripples may underlie the cognitive impairments observed with therapeutic and supratherapeutic doses of CBZ.

## Data Availability

The data that support the findings of this study are available from the corresponding authors T.A.S. and K.A.S. upon reasonable request.

## References

[1] Maan J.S., Duong T.V.H., Saadabadi A. (2020). Carbamazepine. StatPearls [Internet].

[2] Lee S.-A., Lee H.-W., Heo K., Shin D.-J., Song H.-K., Kim O.-J., Lee S.-M., Kim S.-O., Lee B.-I. (2011). Cognitive and behavioral effects of lamotrigine and carbamazepine monotherapy in patients with newly diagnosed or untreated partial epilepsy. Seizure.

[3] Eddy C.M., Rickards H.E., Cavanna A.E. (2011). The cognitive impact of antiepileptic drugs. Ther. Adv. Neurol. Disord..

[4] Forsythe I., Butler R., Berg I., McGuire R. (1991). Cognitive Impairment in New Cases of Epilepsy Randomly Assigned to Carbamazepine, Phenytoin and Sodium Valproate. Dev. Med. Child Neurol..

[5] Fricke-Galindo I., Llerena A., Jung-Cook H., López-López M. (2018). Carbamazepine adverse drug reactions. Expert Rev. Clin. Pharmacol..

[6] Mantegazza M., Curia G., Biagini G., Ragsdale D.S., Avoli M. (2010). Voltage-gated sodium channels as therapeutic targets in epilepsy and other neurological disorders. Lancet Neurol..

[7] West P.J., Saunders G.W., Remigio G.J., Wilcox K.S., White H.S. (2014). Antiseizure drugs differentially modulate θ-burst in-duced long-term potentiation in C57BL/6 mice. Epilepsia.

[8] Buzsáki G. (2015). Hippocampal sharp wave-ripple: A cognitive biomarker for episodic memory and planning. Hippocampus.

[9] Papatheodoropoulos C., Sotiriou E., Kotzadimitriou D., Drimala P. (2007). At clinically relevant concentrations the anaesthet-ic/amnesic thiopental but not the anticonvulsant phenobarbital interferes with hippocampal sharp wave-ripple com-plexes. BMC Neurosci..

[10] Simeone T.A., Simeone K.A., Samson K.K., Kim D.Y., Rho J.M. (2013). Loss of the Kv1.1 potassium channel promotes patho-logic sharp waves and high frequency oscillations in in vitro hippocampal slices. Neurobiol. Dis..

[11] Simeone T., Samson K.K., Matthews S.A., Simeone K.A. (2014). In vivo ketogenic diet treatment attenuates pathologic sharp waves and high frequency oscillations in in vitro hippocampal slices from epileptic Kv1.1α knockout mice. Epilepsia.

[12] Ponomarenko A., Li J.-S., Korotkova T.M., Huston J.P., Haas H.L. (2008). Frequency of network synchronization in the hippocampus marks learning. Eur. J. Neurosci..

[13] Fernández-Ruiz A., Oliva A., Fermino de Oliveira E., Rocha-Almeida F., Tingley D., Buzsáki G. (2019). Long-duration hip-pocampal sharp wave ripples improve memory. Science.

[14] Girardeau G., Benchenane K., Wiener S.I., Buzsáki G., Zugaro M.B. (2009). Selective suppression of hippocampal ripples im-pairs spatial memory. Nat. Neurosci..

[15] Gillespie A.K., Jones E.A., Lin Y.-H., Karlsson M.P., Kay K., Yoon S.Y., Tong L.M., Nova P., Carr J.S., Frank L.M. (2016). Apolipoprotein E4 Causes Age-Dependent Disruption of Slow Gamma Oscillations during Hippocampal Sharp-Wave Ripples. Neuron.

[16] Wiegand J.-P.L., Gray D.T., Schimanski L.A., Lipa P., Barnes C.A., Cowen S.L. (2016). Age Is Associated with Reduced Sharp-Wave Ripple Frequency and Altered Patterns of Neuronal Variability. J. Neurosci..

[17] Sullivan D., Csicsvari J., Mizuseki K., Montgomery S., Diba K., Buzsáki G. (2011). Relationships between hippocampal sharp waves, ripples, and fast gamma oscillation: Influence of dentate and entorhinal cortical activity. J. Neurosci..

[18] Patsalos P.N., Spencer E.P., Berry D.J. (2018). Therapeutic Drug Monitoring of Antiepileptic Drugs in Epilepsy: A 2018 Up-date. Ther. Drug Monit..

[19] Nakashiba T., Buhl D.L., McHugh T.J., Tonegawa S. (2009). Hippocampal CA3 output is crucial for ripple-associated reacti-vation and consolidation of memory. Neuron.

